# Appropriateness of Questionnaires for the Diagnosis and Monitoring Treatment of Dry Eye Disease

**DOI:** 10.3390/jcm13113146

**Published:** 2024-05-27

**Authors:** James S. Wolffsohn, Sònia Travé-Huarte, Jennifer P. Craig, Alex Muntz, Fiona J. Stapleton

**Affiliations:** 1 School of Optometry, College of Health & Life Sciences, Aston University, Birmingham B4 7ET, UK; s.trave-huarte@aston.ac.uk; 2 Department of Ophthalmology, New Zealand National Eye Centre, The University of Auckland, Auckland 1010, New Zealand; jp.craig@auckland.ac.nz; 3 Institute of Optometry, University of Applied Sciences and Arts Northwestern Switzerland, 4600 Olten, Switzerland; 4 School of Optometry and Vision Science, UNSW, Sydney, NSW 2052, Australia; f.stapleton@unsw.edu.au

**Keywords:** dry eye disease, symptom questionnaire, diagnosis, treatment effect, sensitivity, variability, subjective assessment

## Abstract

**Objectives:** If questionnaires contributing to the diagnosis of dry eye disease are to be recommended as alternatives to existing questionnaires, they must be comparable, with similar repeatability and treatment sensitivity. Comparability was thus examined for three common dry eye questionnaires along with identifying the individual questions that most strongly predicted overall scores. **Methods:** Anonymised data (*n* = 329) collected via the Ocular Surface Disease Index (OSDI), 5-item Dry Eye Questionnaire (DEQ-5) and Symptom Assessment in Dry Eye (SANDE) questionnaires (including responses to individual questions) from consenting patients were drawn from real-world dry eye clinics/registries in the United Kingdom, Australia and New Zealand; at follow-up, normalised changes were evaluated in 54 of these patients. Treatment data were also analysed from a 6-month, randomised controlled trial assessing artificial tear supplement treatments with 43 responders and 13 non-responders to treatment identified. The questions extracted from the OSDI which form the abbreviated 6-item OSDI were also analysed. **Results:** The agreement between the questionnaires ranged from r = 0.577 to 0.754 (all *p* < 0.001). For the OSDI, three questions accounted for 89.1% of the variability in the total score. The correlation between the OSDI and OSDI-6 was r = 0.939, *p* < 0.001. For the DEQ-5, two questions accounted for 88.5% of the variance in the total score. Normalised treatment changes were also only moderately correlated between the questionnaires (r = 0.441 to 0.595, *p* < 0.01). For non-responders, variability was 7.4% with both OSDI and OSDI-6, 9.7% with DEQ-5, 12.1% with SANDE-frequency and 11.9% with SANDE-severity scale. For responders, improvement with drops was detected with a 19.1% change in OSDI, 20.2% in OSDI-6, 20.9% in DEQ-5, and 27.5%/23.6% in SANDE-frequency/severity scales. **Conclusions:** Existing commonly used dry eye questionnaire scores do not show high levels of correlation. The OSDI was the least variable of the questionnaires and while displaying a slightly lower treatment effect than either the DEQ or SANDE, it was more sensitive to detection of a treatment effect. The quicker-to-complete OSDI-6 exhibited essentially the same outcome as the OSDI, with similar variability and treatment sensitivity.

## 1. Introduction

Dry eye disease is a subset of ocular surface disease, with its definition requiring the presence of both clinical signs and patient-reported symptoms [1,2]. The Tear Film and Ocular Surface Society (TFOS) Dry Eye Workshop II [3] identified the Ocular Surface Disease Index (ODSI) and the 5 item Dry Eye Questionnaire (DEQ-5) as the most robustly designed, evaluated and utilised questionnaires in dry eye disease evaluation and, therefore, recommended the use of either one for the symptoms element of the diagnosis of dry eye disease [4,5]. However, a comparison of the unified outcomes of these questionnaires had not been assessed at that time, and poor comparability of questionnaires has since been identified [6]. There are a wide range of questionnaires that have been developed for or used in studies for dry eye disease [7,8,9,10,11,12,13,14,15,16,17,18]. Reviews of all currently available dry eye questionnaires identified that none met modern standards of questionnaire design guidelines [19,20].

Recent papers comparing these two questionnaires (OSDI and DEQ-5) reported a correlation of 0.649 in a population of 392 in Ghana (but over one-third were asymptomatic of dry eye) [21], and a correlation of 0.566 in a population of 101 in India [22]. This leads to uncertainty in this element of dry eye disease diagnosis in some patients [23,24]. The Symptom Assessment iN Dry Eye (SANDE) questionnaire, with its global assessment of dry eye frequency and severity reported on a visual analogue scale, also provides a sensitive (68%) and specific (94%) test in combination with non-invasive tear breakup time as an alternative to assessing the full TFOS DEWS II recommended diagnosis of dry eye disease [25].

The OSDI, with its 12 questions, places a response burden on patients, so a study was conducted confirming it could be shortened without significantly impacting the outcome; the resulting abbreviated OSDI-6 was more repeatable than either the full OSDI or DEQ-5 [26]. In addition, the scoring was able to be simplified to the sum of the item scores, with a diagnostic cut-off of ≥3 for dry eye disease symptomology, making it easier for clinicians to quickly evaluate [26]. This shortened form may also prove easier for children to complete, as less time and assistance are required to complete shorter questionnaires in this population [27].

Hence, this study compared the association between three questionnaires, the OSDI, DEQ-5 and SANDE, in patients attending dry eye clinics in the United Kingdom, Australia and New Zealand, to assess their comparability, repeatability and treatment sensitivity and to identify the individual questions most strongly predicting the overall scores.

## 2. Materials and Methods

De-identified data on the OSDI, DEQ-5 and SANDE questionnaires completed at the same visit (including responses to individual questions) from 329 consenting patients were drawn from real-world dry eye clinics/patient registries in the United Kingdom (Aston University Eye Clinic), Australia (University of New South Wales Optometry Clinic) and New Zealand (University of Auckland Ocular Surface Registry). The questions extracted from the OSDI which form the abbreviated 6-item OSDI were also analysed (see Table 4) [26]. Fifty-four of the patients had follow-up data from each of the questionnaires post treatment which were used to assess whether the questionnaire’s total scores changed by a similar proportion between visits.

To assess the questionnaires’ treatment sensitivity, separate data from 56 patients were analysed from a 6-month, randomised controlled trial assessing artificial tear supplement therapy conducted at the authors’ institutions [28]. This study involved a baseline clinical assessment, prescription of one of two artificial tears (randomized across sites) for use four times a day and follow-ups at monthly visits over six months (a total of seven visits) at which the OSDI, DEQ-5 and SANDE questionnaires were completed.

In all cases, the questionnaires were self-completed sequentially on paper/electronically to avoid score inflation from supported completion [29].

To detect a 0.2 difference in correlation with 80% power and desired significance of *p* < 0.05, a sample size of 318 (G*Power v3.1.9.6) was required. For dependent variables (change), a small effect size (Cohen = 1) could be detected with a sample size of 27. 

### Statistical Analysis

Data were predominantly ordinal, arising from Likert scales; therefore, they were treated as non-parametric data for statistical analysis using SPSS v28. Spearman’s rank correlation testing assessed association between questionnaires, and linear multivariate modelling (forward stepwise approach) was used to assess predictive ability of individual items to reflect the questionnaire outcomes as a whole (which is how they are normally interpreted). The questionnaires were scaled to a 100-point range to allow a Bland–Altman analysis of change between visits, variability of treatment in non-responders (taken as the standard deviation between visits) and treatment effect (the change from baseline of the average score from 1 to 6 months post treatment). 

## 3. Results

Data for all three questionnaires were available from 329 individuals examined since 2018 (median age 42 years, range 20–86 years; 34% male; 56% White, 35% Asian and 9% of other ethnicities). The OSDI had a median score of 31.1 (range 0 to 100, 25th quartile 22.9 and 75th quartile 56.8). The DEQ-5 had a median score of 13.0 (range 0 to 21, 25th quartile 10.0 and 75th quartile 15.0). The SANDE had a median frequency score of 65 (range 0 to 100, 25th quartile 44 and 75th quartile 83) and severity score of 55 (range 0 to 100, 25th quartile 35 and 75th quartile 74).

### 3.1. Association between Questionnaires and Predictive Items

The pair-wise correlation between the questionnaires ranged from 0.577 to 0.754 (all *p* < 0.001; Table 1).

- For the OSDI, question 11 (low humidity areas) accounted for 64.5% of the variance in overall score, 82.4% with the addition of question 9 (watching television), 89.1% with question 4 also added (blurred vision) and 92.2% complete with question 3 (painful or sore eyes). Each of the 12 questions individually strongly correlated with the final score (r ≥ 0.539, *p* < 0.001) and moderately correlated with each other (r ≥ 0.177, all *p* < 0.001; Table 2).

Summing up the responses for the questions that form the OSDI-6, the correlation with the full OSDI was 0.939, *p* < 0.001.

For the DEQ-5, question 2 (discomfort severity) accounted for 75.7% of the variance in overall score, increasing to 88.5% with the addition of question 3 (dryness frequency) and 95.9% with further addition of question 5 (watery eyes). The first four questions (frequency and severity of discomfort and dryness) strongly correlated with the final score (r > 0.740, *p* < 0.001), whereas the association with question 5 (watery eyes) resulted only in r = 0.320 (*p* < 0.001); the strongest correlation for question 5 with questions 1 to 4 was just r = 0.108 (*p* > 0.05).

### 3.2. Association between Questionnaires in Evaluating Change

**Figure 1:**
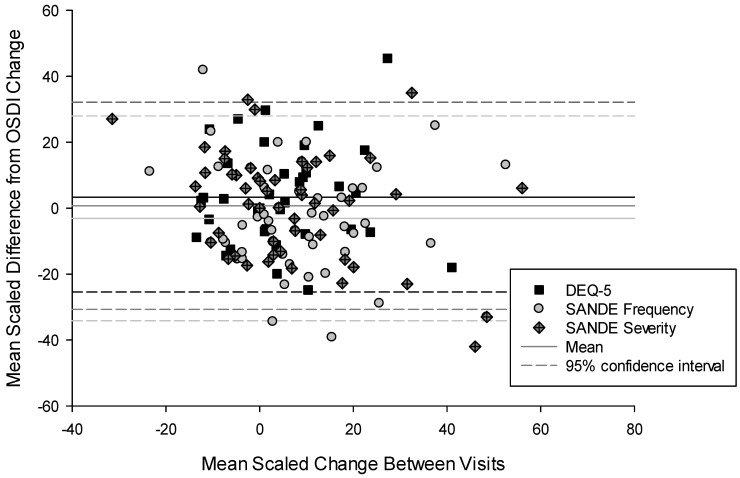
Bland–Altmann scaled difference compared to the mean in Ocular Surface Disease Index (OSDI) change to that of the 5-item Dry Eye Questionnaire (DEQ-5) and Symptom Assessment iN Dry Eye (SANDE) questionnaire scales between visits, showing the lack of consistency within an individual. *n* = 54. Solid line = mean. Dashed line = 95% confidence interval (1.96 × standard deviation).

Fifty-four of the original cohort participants contributed follow-up data from each of the questionnaires post-treatment. To determine how comparable the questionnaires were at evaluating change in an individual, the difference between visits was scaled to a percentage of the scale range, and the results for each questionnaire underwent correlation analysis (Table 3). While the mean difference from the OSDI change was small (within 4%), the variability between individuals was large (29 to 31%; Figure 1).

### 3.3. Treatment Sensitivity

Analysing the data from the artificial tear treatment study, responders to treatment were required to exhibit a drop in OSDI of at least 7.3 (the upper end of the meaningful clinically important difference for mild to moderate dry eye disease) [30]. Based on this criterion, *n* = 30 were non-responders to drops (previously reported) [28]. Requiring a drop in DEQ-5 of at least 1.5, *n* = 29 were non-responders to drops (79% agreement with OSDI non-responders). Requiring a drop in SANDE frequency of at least 4, *n* = 24 were considered non-responders to drops (78% agreement with OSDI and 80% with DEQ-5 non-responders). Requiring a drop in SANDE severity of at least 4, *n* = 29 were deemed non-responders to drops (75% agreement with OSDI and 76% with DEQ-5 non-responders). All questionnaires offered agreement in regard to an individual being classified as a responder in *n* = 43, and there were 13 participants for whom all questionnaires consistently identified them as a non-responder.

For non-responders, variability (the standard deviation across all 7 visits) was 7.4% with OSDI, 9.7% with DEQ-5, 12.1% for SANDE-frequency and 11.9% for SANDE-severity scale. Variability was highest with questions 11 (low humidity), question 12 (air conditioning), question 8 (bank machine) and question 3 (painful or sore eyes) for the OSDI. For the DEQ-5, questions relating to severity (both standard deviations = 0.74) were more variable than those relating to frequency (0.45 and 0.54; Wilcoxon test *p* = 0.004). Summing just the OSDI-6 questions, the variability was 7.4%.

For responders, improvement with drops was 19.1% according to the OSDI, 20.9% for the DEQ-5 and 27.5% and 23.6% on the SANDE-frequency and SANDE-severity scales, respectively. The items driving the greatest change in the OSDI were question 12 (air conditioning), question 10 (windy conditions) and question 3 (painful or sore eyes). For the DEQ-5, questions relating to dryness demonstrated insignificantly more change than those relating to discomfort (*p* = 0.184), with much less change in the ‘watery eyes’ item (*p* < 0.001); summing solely the OSDI-6 questions, the improvement with drops was found to be 20.2% in the study population.

## 4. Discussion

This study compared the association between three questionnaires, the OSDI, DEQ-5 and SANDE, completed at the same visit by consenting patients attending dry eye clinics in the United Kingdom, Australia and New Zealand, to assess their comparability, repeatability and treatment sensitivity along with identifying which individual questions were the strongest predictors of the overall scores. As found previously [21,22], the results generated from self-completion of the most commonly administered dry eye disease questionnaires are only modestly correlated. Hence, recommending either one or the other to be used as part of the diagnosis of dry eye disease will lead to a lack of consistency. Assessment of how individual items are associated with the overall score demonstrates that many of the items within the OSDI are redundant, unnecessarily increasing the response burden for patients. While each of the items of the OSDI examine the same trait (all questions significantly correlated with the composite score (r > 0.5, *p* < 0.001), and each other (*p* < 0.01), this was not the case for the DEQ-5 where the final question on watery eyes did not correlate with any of the other items. However, watery eyes were reported to occur by 78.4% of the patients, but only in 1.0% as the sole DEQ-5 symptom, thus making this question largely redundant. The correlation between the OSDI and SANDE was found to be similar to that reported during the SANDE questionnaire development (r = 0.64) [31] and subsequent evaluations (r = 0.53 [32]; r = 0.59 [33]). However, this translated to only 28 to 41% of the variance in the OSDI being accounted for, challenging its ability “to provide clinicians with a short, quick, and reliable measure for dry eye disease symptoms” [31], although it has been shown to have a high sensitivity and specificity to a TFOS DEWS II diagnosis of dry eye disease [25].

Normalising the questionnaire ranges allowed comparison of the reliability of the questionnaires in evaluating change. The correlations between questionnaires were again only moderate as has been found in a similar analysis between the OSDI and SANDE (r = 0.47 [31]; r = 0.63 [33]). Using data from a treatment study with clear responders and non-responders [28], the discord between the questionnaires was again evident. The variability in scores for non-responders was lowest for the OSDI and highest for the SANDE scales. This followed a similar pattern to sensitivity to treatment (with questions being more sensitive to treatment also being those that were generally more variable in non-responders); however, a standard Wilcoxon or Mann–Whitney test to detect the treatment effect compared to a comparator would require 55% more participants with the DEQ-5, 29% with the SANDE-frequency scale, and 70% with the SANDE-severity scale than the OSDI (G*Power). The top three predictive items for the total OSDI score found in this study are included within the OSDI-6 (along with three others so all domains are covered; Table 4), resulting in a correlation between the OSDI and OSDI-6 (r = 0.94) which confirms the results reported during its development [26]. In addition, this study shows the variability of the OSDI-6 to be equivalent to that of the original OSDI, but the treatment effect was measured to be larger, suggesting that a study using the OSDI-6 would require 11% fewer participants than one using the OSDI for matched statistical power. 

This study used a large sample of data, encompassing individuals reporting ‘no’ to extreme symptoms, who completed the questionnaires at the same time to negate variations in recollection. The study involved secondary analysis of the data,, but typical of the real-world and multi-national. While the use of these questionnaires aim to establish symptomatology status during the presence of the dry eye disease (true positives), a limited number of non-dry-eye participants (true negatives) were included in this study to assess the diagnostic ability of the questionnaires. Questionnaires used in isolation are limited in ‘their ability to diagnose’ dry eye disease as a differential diagnosis is needed to exclude conditions which can result in similar symptoms (such as allergy and infection) [4]. The diagnosis of dry eye, by definition, also requires both clinical signs and symptoms to be present [2]. In those diagnosed with dry eye disease, a subclassification of the type of dry eye a patient is experiencing is also required to inform the most suitable management approach [4,34,35].

In conclusion, all of the questionnaires were able to differentiate a treatment effect and, so, are valid for use in dry eye research. However, these commonly used current dry eye questionnaires are not interchangeable so there is a need to have only one recommended in order to create robust diagnostic criteria. The OSDI is the least variable of the questionnaires, and while finding a slightly lower treatment effect than the DEQ-5 or SANDE, it is more sensitive at detecting a treatment effect. The OSDI-6 produces essentially the same outcome as the OSDI, takes less time to complete than the OSDI, has similar variability and a better treatment sensitivity and, therefore, is worthy of consideration as a replacement questionnaire for dry eye diagnostic purposes.

## Figures and Tables

**Table 1:** Correlation between the Ocular Surface Disease Index (OSDI), 5-item Dry Eye Questionnaire (DEQ-5) and Symptom Assessment iN Dry Eye (SANDE) questionnaires. *n* = 329; shaded cells—comparisons are not needed as already covered in the scale.

SPEARMAN’S r	OSDI	DEQ-5	SANDE Frequency
DEQ-5	0.577		
SANDE Frequency	0.565	0.572	
SANDE Severity	0.626	0.650	0.754

**Table 2:** Correlation between each of the Ocular Surface Disease Index (OSDI) questions and those comprising the OSDI-6. *n* = 329. All *p* < 0.001; shaded cells—comparisons are not needed as already covered in the scale.

SPEARMAN’s r	Gritty	Sore	Blurred Vision	Poor Vision	Reading	Driving at Night	Using Computer	Watching Television	Windy Conditions	Low Humidity	Air Conditioning	*OSDI TOTAL*	*OSDI-6 TOTAL*
Light sensitivity	0.376	0.360	0.359	0.288	0.332	0.377	0.278	0.316	0.388	0.305	0.304	***0.573***	***0.637***
Gritty		0.562	0.374	0.260	0.340	0.342	0.325	0.372	0.408	0.468	0.432	***0.631***	** **
Sore			0.312	0.232	0.483	0.443	0.525	0.518	0.391	0.537	0.476	***0.700***	** **
Blurred vision				0.682	0.411	0.396	0.351	0.396	0.264	0.302	0.286	***0.586***	***0.568***
Poor vision					0.419	0.399	0.324	0.356	0.177	0.317	0.299	***0.539***	** **
Reading						0.546	0.739	0.655	0.359	0.490	0.450	***0.739***	** **
Driving at night							0.570	0.591	0.408	0.456	0.429	***0.723***	***0.746***
Using computer								0.731	0.333	0.516	0.431	***0.724***	** **
Watching television									0.383	0.518	0.472	***0.746***	***0.705***
Windy conditions										0.717	0.667	***0.683***	***0.756***
Low humidity											0.825	***0.793***	***0.782***
Air conditioning												***0.741***	** **
***OSDI TOTAL***												** **	***0.939***

**Table 3:** Correlation between the percentage change in Ocular Surface Disease Index (OSDI), 5-item Dry Eye Questionnaire (DEQ-5) and Symptom Assessment iN Dry Eye (SANDE) questionnaires following treatment. *n* = 54. All *p* < 0.01; shaded cells—comparisons are not needed as already covered in the scale.

SPEARMAN’S r	OSDI	DEQ-5	SANDE Frequency
DEQ-5	0.463		
SANDE Frequency	0.441	0.483	
SANDE Severity	0.549	0.436	0.595

**Table 4:** The Ocular Surface Disease Index 6-item (OSDI-6). A summed score ≥ 3 indicates a positive symptom score that contributes to the diagnosis of dry eye disease Adapted from [26].

	Constantly	Mostly	Often	Sometimes	Never
**Have you experienced any of the following *during a typical day within the last month?***					
1. Eyes that are sensitive to light?	4	3	2	1	0
2. * Vision blurring between blinks, with your refractive correction *?	4	3	2	1	0
	Poor symptoms and visual disturbance subscale score ⇨				
**Have problems with your eyes limited you in performing any of the following *during a typical day within the last month?***					
3. Driving or being driven at night?	4	3	2	1	0
4. Watching TV, or a similar task?	4	3	2	1	0
	Visual function/tasks subscale score ⇨				
**Have your eyes felt uncomfortable in any of the following situations *during a typical day within the last month?***					
5. Windy conditions?	4	3	2	1	0
6. Places or areas with low humidity?	4	3	2	1	0
	Environmental subscale score ⇨				

* suggested adaptation to improve the comprehension of this question.

## Data Availability

The data presented in this study are available on request from the corresponding author.
